# Role of Procalcitonin and Interleukin-6 in Predicting Cancer, and Its Progression Independent of Infection

**DOI:** 10.1371/journal.pone.0130999

**Published:** 2015-07-06

**Authors:** Anne-Marie Chaftari, Ray Hachem, Ruth Reitzel, Mary Jordan, Ying Jiang, Ammar Yousif, Kumait Garoge, Poonam Deshmukh, Zanaib Al Hamal, Joseph Jabbour, Alexander Hanania, Sammy Raad, Mohamed Jamal, Issam Raad

**Affiliations:** Department of Infectious Diseases, Infection Control and Employee Health/Division of Internal Medicine, The University of Texas M.D. Anderson Cancer Center, Houston, United States of America; Peking University Cancer Hospital & Institute, CHINA

## Abstract

Procalcitonin (PCT) and Interleukin-6 (IL-6) have emerged as biomarkers for different inflammatory conditions. The purpose of the study was to evaluate the role of PCT and IL-6 as biomarkers of cancer and its progression in a large cohort of patients. This cross-sectional study included residual plasma samples collected from cancer patients, and control subjects without cancer. Levels of PCT and IL-6 were determined by Kryptor compact bioanalyzer. We identified 575 febrile cancer patients, 410 non-febrile cancer patients, and 79 non-cancer individuals. The median PCT level was lower in control subjects (0.029 ng/ml) compared to cancer patients with stage I-III disease (0.127 ng/ml) (p<0.0001) and stage IV disease (0.190 ng/ml) (p<0.0001). It was also higher in febrile cancer patients (0.310 ng/ml) compared to non-febrile cancer patients (0.1 ng/ml) (p<0.0001). Median IL-6 level was significantly lower in the control group (0 pg/ml) than in non-febrile cancer patients with stages I-III (7.376 pg/ml) or stage IV (9.635 pg/ml) (p<0.0001). Our results suggest a potential role for PCT and IL-6 in predicting cancer in non-febrile patients. In addition, PCT is useful in detecting progression of cancer and predicting bacteremia or sepsis in febrile cancer patients.

## Introduction

Procalcitonin (PCT) is the prohormone of calcitonin and thought to be mainly produced in the liver by macrophages (Kupffer cells) or neuroendocrine cells [1–3]. Over the last two decades several studies have demonstrated that PCT is highly associated with bacterial infections, particularly bacteremia [4–6]. PCT has been suggested as a potential biomarker for sepsis and infection and as a guide to antibiotic administration [5, 7].

Neuroendocrine tumors may exhibit high levels of serum PCT and an association has been determined between PCT levels and the clinical course of these tumors [8]. Matzaraki et al. also reported increased serum PCT levels in cancer patients with metastatic disease [9]. The role of PCT in febrile cancer patients (with possible infection) has been previously evaluated by our group, in separate studies that included non-neutropenic solid tumor patients[5] as well as patients with hematological malignancies whom many were neutropenic [6], and was found to be a predictor of sepsis and bloodstream infections.

Interleukin-6 is a cytokine growth factor that stimulates cell growth and up regulates the acute phase response proteins associated with inflammation and injury [10]. Like PCT, IL-6 is thought to be produced in the Kupffer cells of the liver [11]. More recently, several studies have shown that IL-6 is also expressed in the cytoplasm of different cancer cells [12–14]. Furthermore, a study suggested that advanced cancer patients with liver metastasis have higher levels of IL-6 [9].

However, few studies evaluated the role of PCT and IL6 in predicting cancer and its progression independent of co-existing infection [9]. The purpose of this current study was to evaluate the role of PCT and IL-6 as biomarkers of cancer in a large cohort of non-febrile cancer patients, to determine their diagnostic effectiveness in the prediction of cancer progression and metastasis, and to compare the levels of those biomarkers to non-cancer control patients. Furthermore, because PCT is a known biomarker of infection we compared PCT levels in non-febrile cancer patients to febrile cancer patients in order to determine whether baseline PCT level in cancer patients further increases in the setting of infection. Our hypothesis was that in the absence of infections, cancer patients, particularly those with advanced stages such as metastasis, have higher PCT and IL-6 levels than those with no evidence of cancer. However, infections (particularly sepsis and bloodstream infections) could further increase the PCT level.

## Materials and Methods

We conducted a cross-sectional clinical laboratory study that included non-febrile and febrile cancer patients who presented to The University of Texas MD Anderson Cancer Center between August 2009 and November 2009 for which we were able to obtain residual plasma sample after it has been used for routine clinical chemistry. Febrile patients presented with a temperature of ≥38.3°C or 2 consecutive readings >38°C. within 24 hours of blood sample collection. Sepsis was defined as the presence of 2 or more of the following conditions: temperature >38.5°C or <35°C, heart rate >90 beats/minute, respiratory rate >20 breaths/minute and white blood cell count (WBC) >12 000 cells/mL, <4000 cells/mL, or >10% immature (band) forms in the presence of a microbiologically documented infection (culture or Gram stain of blood, sputum, urine, or normally sterile body fluid positive for a pathogenic microorganism) [15, 16]. Bacteremia was defined by positive blood cultures associated with fever as a clinical manifestation of infection. Neutropenia was defined as an absolute neutrophil count (ANC) of <500 cells/mm^3^ according to the Infectious Diseases Society of America 2011 clinical practice guideline for the use of antimicrobial agents in neutropenic patients with cancer [17]. Non-febrile cancer patients had no evidence of fever as defined above and no signs and symptoms suggestive of infections at time of blood sample collection. Control subjects included healthy volunteers who worked at MD Anderson Cancer Center during the same period and patients who presented for screening at our institution with no evidence of cancer. This study was approved by the institutional review board (IRB) of The University of Texas M. D. Anderson Cancer Center (Houston, TX). Since this study involved collection of a residual sample and chart review, the IRB specifically waived the need for written informed consent from the patients. However, a signed written informed consent was obtained from the healthy volunteers per IRB recommendation.

Electronic medical records for each patient were reviewed for data collection including demographic data, admitting diagnosis, date of admission, and underlying cancer and its stage. Clinical information pertinent to the presence of infection was also collected including vital signs, neutropenia, radiologic imaging findings, and microbiologic data.

PCT levels were measured using Kryptor compact bioanalyzer (ThermoFisher, Waltham, MA). Acceptable PCT levels were defined to be within the reading limits of the bioanalyzer (0.75mg/mL lower limit; >50ng/mL upper limit), any sample that exceeded the upper limit was automatically by the machine diluted for retest [18].

Plasma IL-6 concentrations were measured only in non-febrile cancer patients as well as non-cancer patients using a quantitative sandwich enzyme-linked immunosorbent assay (ELISA) according to the instructions of the manufacturer (eBioscience, Inc., San Diego, CA). The calibrations on each microtiter plate included recombinant human IL-6 standards. Optical density was determined using a microtiter plate reader (Thermo Scientific, Inc., Hudson, NH) at 450nm and concentrations were reported as pg/ml. Plasma concentrations were determined by a lab personal who was blind to clinical history of the patients.

### Statistical methods

Chi-square or Fisher’s exact tests were used to compare categorical variables, as appropriate. Wilcoxon rank sum tests were used to compare continuous variables, including comparing PCT or IL-6 levels between two groups of patients. For multiple-group comparisons, PCT or IL-6 levels were first compared using Kruskal-Wallis test. If a significant result (*p*<0.05) was detected, then Wilcoxon rank sum tests were used for pairwise comparisons. The α levels of the post-hoc pairwise comparisons were adjusted using a sequential Bonferroni adjustment. Spearman’s rank correlation was assessed between patients’ PCT levels and IL-6 levels. To further evaluate the association of PCT level with factors such as cancer, fever and bacteremia or sepsis, multivariate analysis was performed using generalized linear model (GLM). Given that PCT data were non-parametric data, rank transformation was performed before GLM analysis. GLM method was also used in subset analysis, including the analysis in cancer patients, and the analysis in cancer patients with solid tumor or lymphoma only. Besides, the performance of PCT test to differentiate febrile cancer patients with bacteremia or sepsis versus non-febrile cancer patients was assessed. First, a receiver operating characteristic (ROC) curve analysis was performed. The area under the ROC curve (AUC) and its 95% confidence interval were estimated. Then the optimal cut-off point for the test was determined on the basis of its ROC curve. Lastly, the associated sensitivity, specificity, and positive and negative predictive values were estimated. All tests except those for pairwise comparisons were two-sided tests with a significance level of 0.05. The statistical analyses were performed using SAS version 9.3 (SAS Institute Inc., Cary, NC).

## Results

### Patients’ demographic and clinical data

**Fig 1:**
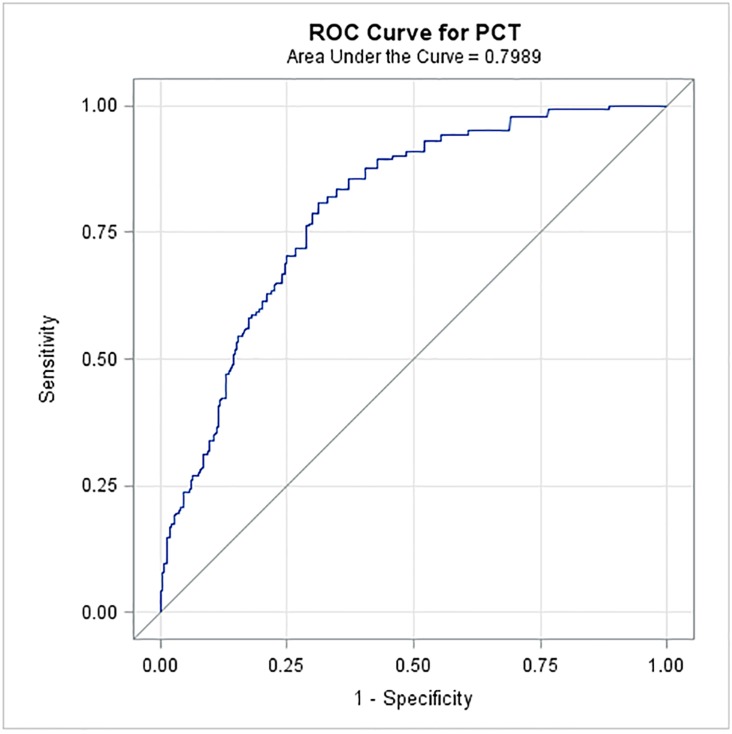
Patients Flow Chart.

We evaluated 1,064 patients that included 575 febrile and 410 non-febrile cancer patients, as well as 79 non-cancer individuals (Fig 1). The demographic and clinical characteristics of the non-febrile and febrile cancer patients are shown in Table 1. Cancer patients with fever were more likely to have hematological malignancies (66% vs 26%, *p*<0.0001) and be neutropenic (35% vs. 9% respectively; *p*<0.0001) compared to non-febrile cancer patients.

**Table 1:** Demographic and clinical characteristics of the cancer patients.

Characteristic	Fever (n = 575) N (%)	non-Fever	*p*-value
Age (year), median (range)	57 (2–92)	(n = 410)	0.1
		N (%)	
Gender, male	322 (56%)	231 (56%)	0.92
Neutropenia at onset	203 (35%)	36/408 (9%)	< .0001
Underlying cancer			< .0001
Hematologic malignancy	381 (66%)	107 (26%)	
Solid tumor	194 (34%)	303 (74%)	
Commonest type of cancer			
Breast cancer	11 (1.9%)	15 (3.7%)	
Lung cancer	12 (2.1%)	32 (7.8%)	< .001
Prostate cancer	4 (0.7%)	27 (6.6%)	
Colon cancer	27 (4.7%)	22 (5.4%)	0.63
Sarcoma cancer	17 (3.0%)	31 (7.6%)	0.001
Brain cancer	10 (1.7%)	16 (3.9%)	
Leukemia	228 (39.7%)	44 (10.7%)	< .0001
Lymphoma	87 (15.1%)	55 (13.4%)	0.45
Thyroid cancer	3 (0.5%)	5 (1.2%)	
Other types of cancer	176 (30.6%)	163 (39.8%)	
Cancer stage of patients with solid tumor or			0.13
lymphoma			
Stage I, II, and III	66/212 (31%)	120/320 (38%)	
Stage IV	146/212 (69%)	200/320 (62%)	
NA or unknown	69	32	
Status of cancer			< .0001
Active	487/569 (86%)	391 (95%)	
Remission	82/569 (14%)	19 (5%)	
Unknown	6		
For patients with active cancer			< .0001
New diagnosis	170/459 (37%)	117/391 (30%)	
Relapsed	119/459 (26%)	52/391 (13%)	
Refractory/progression/Metastasis	170/459 (37%)	222/391 (57%)	
Unknown	28		
Active treatment	495/571 (87%)	354 (86%)	0.87

#### PCT levels

In cancer patients, the PCT levels varied according to cancer type. It was found that the median PCT was higher in patients with hematological malignancies than those with solid tumors (0.230 ng/ml [range 0–129.3] vs. 0.156 ng/ml [range 0–154.7]; *p* <0.0001). In the commonest types of malignancies, the median PCT was found to be high in colon cancer (0.400 ng/ml [range 0–81.95]), leukemia (0.265 ng/ml [range 0–129.3]), thyroid cancer (0.231 ng/ml [range 0.034–13.51]), lymphoma (0.165 ng/ml [range 0–67.38]) prostate cancer (0.164 ng/ml [range 0–2.87]), and sarcoma (0.147 ng/ml [range 0–24.67]). It was significantly higher in patients with leukemia and colon cancer compared to patients with breast cancer (0.073 ng/ml, [range 0–9.64]) (*p* = 0.001 and *p* = 0.01 respectively).

In the control group of non-cancer patients the median PCT level (0.029 ng/ml [range 0–0.203]) was lower than in cancer patients with stage I-III disease (0.127 ng/ml [range 0–18.13]; *p*<0.0001) or those with advanced stage IV (0.190 ng/ml [range 0–154.7]; *p*<0.0001). Among cancer patients, those who had advanced stage IV had a higher median PCT levels than those with stage I-III (0.190 ng/ml [range 0–154.7] vs. 0.127 ng/ml [range 0–18.13]; *p* = 0.004) (Table 2).

**Table 2:** Comparing PCT and IL-6 among solid tumor and lymphoma patients with different cancer stages.

	Non-cancer	Cancer stage I, II and III	Cancer stage IV	*p*-value
Median PCT (ng/ml), (range)	0.029 (0–0.203)	0.127 (0–18.13)	0.190 (0–154.7)	< .0001
	(n = 79)	(n = 186)	(n = 346)	
Median IL-6 (pg/ml), (range)	0 (0–211.9)	7.376 (0–200.8)	9.635 (0–307.7)	< .0001
	(n = 73)	(n = 120)	(n = 206)	

Note:

1) IL-6 test was performed in non-febrile patients only while PCT test was performed in both febrile and non-febrile patients.

2) All pairwise comparisons for PCT have a *p* < 0.001.

3) All pairwise comparisons for IL-6 have a *p*<0.0001 except the comparison between the patients with cancer stages I, II, and III vs IV (*p* = 0.2).

**Fig 2:**
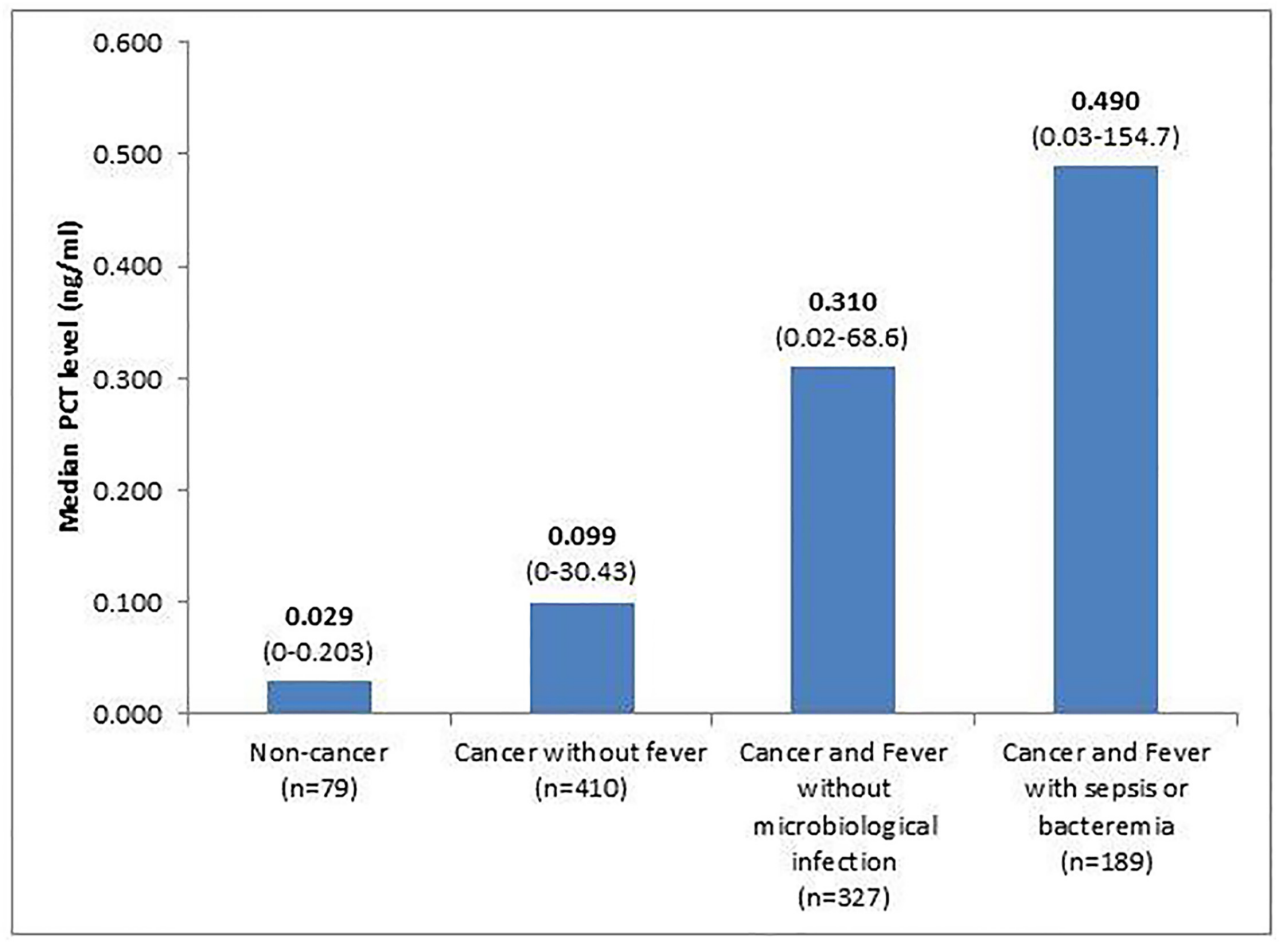
Comparing PCT among different groups of patients.

**Fig 3:**
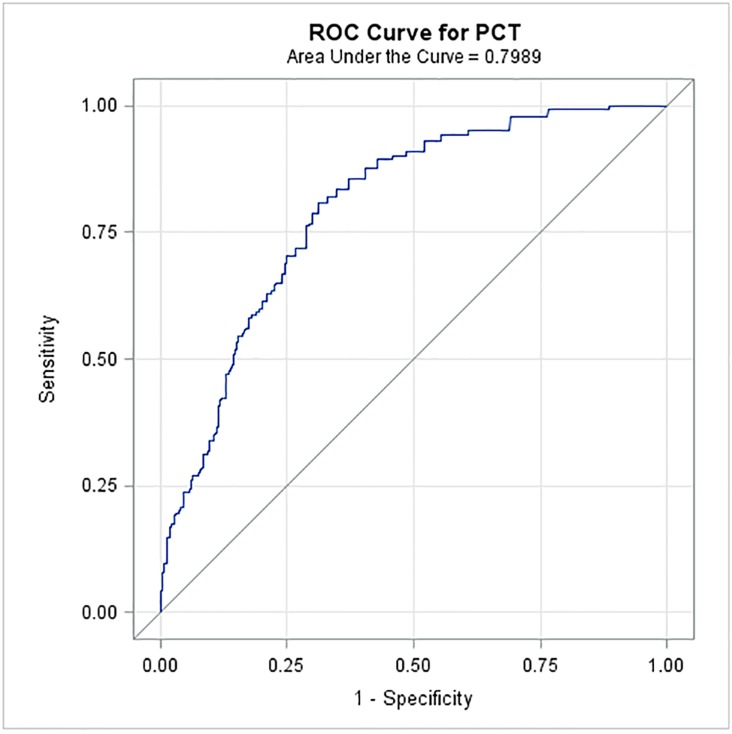
ROC curve of PCT test to differentiate febrile cancer patients with bacteremia or sepsis versus non-febrile cancer patients.

The median PCT level was higher in febrile cancer patients (0.310 ng/ml [range 0.02–154.7]) compared to non-febrile cancer patients (0.1 ng/ml [range 0–30.43]) as well as to the control group of non-cancer patients (0.029 ng/ml [range 0–0.203]) (*p*<0.0001). We further compared PCT level among different groups of patients (Fig 2). Specifically, non-cancer patients had a significantly lower PCT level (median: 0.029 ng/ml) than non-febrile cancer patients (median: 0.099 ng/ml) (*p*<0.0001) and the latter had a significantly lower PCT level than febrile cancer patients without microbiological infection (median: 0.310 ng/ml) (*p*<0.0001). Among the febrile cancer patients, those without microbiological infection had a significantly lower PCT level than those who had sepsis or bacteremia (median: 0.490 ng/ml) (*p* = 0.003) (Fig 2). An ROC analysis was performed for the PCT test to differentiate febrile cancer patients with bacteremia or sepsis versus non-febrile cancer patients. The area under the ROC curve was 0.80 with a 95% confidence interval of 0.76 to 0.83 (Fig 3). The optimal cut-off point was 0.17 ng/ml, with a sensitivity of 81%, a specificity of 69%, a positive predictive value of 54%, and a negative predictive value of 89%.

The association of PCT levels with the factors described above was further evaluated in multivariate analysis using generalized linear model (GLM) to adjust for possible confounders. The factors we considered for all patients included cancer, fever, bacteremia or sepsis, and active treatment. The analysis showed that each of the following three factors was independently associated with increased PCT level: cancer (*p*< .0001), fever (*p*< .0001) and bacteremia or sepsis (*p* = 0.0003). In cancer patients, a GLM analysis showed no significant association between PCT level and cancer type (hematologic malignancy vs solid tumor) (*p* = 0.20) after adjusting for fever (*p* < .0001) and bacteremia or sepsis (*p* = 0.0005). In cancer patients with solid tumor or lymphoma, another GLM analysis showed that cancer stage (*p* = 0.013) and fever (*p*< .0001) were the only factors independently associated with PCT level, confirming that patients with cancer stage IV had increased PCT level compared to patients with stage I-III.

#### IL-6 levels

IL-6 levels were measured in control patients and non-febrile cancer patients. A non-parametric correlation test showed a moderate positive correlation between IL-6 and PCT levels in these patients (correlation coefficient = 0.41, *p*< .0001). The median IL-6 level in the control group was significantly lower than in patients with low stages of cancer or advanced stage IV cancer (0 pg/ml vs 7.376 pg/ml and 9.635 pg/ml, respectively, *p*<0.0001) (Table 2). However, the median IL-6 level in patients with advanced stage IV cancer was not significantly different than in patients with stage I, II and III.

## Discussion

In this study, we found that PCT and IL-6 could have a role in predicting cancer as our findings showed that PCT and IL-6 levels were higher in cancer patients than normal subjects. However, PCT was especially higher in those with advanced stage of the disease and may play a role in assessing progression of the disease. PCT was also high in certain malignancies and reached the highest levels in febrile cancer patients particularly those who have documented bloodstream infections and sepsis.

Our findings of higher PCT levels in certain malignancies such as: colon cancer, leukemia, thyroid cancer, lymphoma, and prostate cancer agree with previous reports of increased serum PCT in tumors such as small cell lung cancer, carcinoid, pheochromocytoma, pancreatic islet, breast, thyroid, lung, gastro-intestinal including colon cancer and urogenital tumors [8, 9, 19, 20]. We also found that PCT levels were higher in patients with hematological malignancies. A study of 65 patients with chemo-induced neutropenia showed similar finding to ours in that PCT levels were higher in patients with hematological malignancies than patients with solid tumors and especially higher in patients with leukemia compared to patients with breast cancer [21]. However, in a multivariate analysis, after adjusting for fever and bacteremia or sepsis, there was no significant association between PCT level and cancer type (hematologic malignancy vs solid tumor). This could be related to the higher tendency of patients with hematologic malignancy to be febrile and be prone to infections.

In addition, the finding of higher levels of PCT in metastatic advanced diseases is similar to what has been published in literature. In a study involving 54 patient with colon cancer, patients with distant metastases had higher serum PCT levels than patients without distant metastases [19]. Another study of 43 solid tumors cancer patients showed that serum PCT levels in patients with generalized metastatic carcinoma were significantly higher than serum PCT in control healthy patients and control cancer patients without metastasis [9].

Another finding in this study is that PCT was helpful in predicting bacteremia or sepsis in febrile cancer patients as it has been markedly higher in patients with these documented infections. This is similar to our findings in other studies of patients with hematological malignancies and non-neutropenic cancer patients where PCT was higher in patients with documented bacteremia or sepsis [5, 6]. Other studies in febrile neutropenic patients had similar findings to ours in that PCT was a good marker of documented bloodstream infections [22–24]. In another small study, involving 37 febrile episodes in patients with urological cancers, procalcitonin levels were significantly higher in episodes with documented bacterial infections than those without [25].

In this current study, the cut-off value for PCT to differentiate patients with bacteremia or sepsis versus those without infection is lower than what has been published in a study in immunocompromised patients of > 0.5 ng/ml with 100% sensitivity but only 63% specificity for diagnosing bacterial sepsis [26] and another of 2 ng/ml with 75% specificity and 90% sensitivity [27].

Similarly, our study have shown that IL-6 was also elevated in patients with cancer compared to the control group, which support previous studies where IL-6 is expressed in the cytoplasm of glioblastoma, breast, renal, bladder, prostate and cervical cancer cells [12–14]. Several experimental studies have suggested that IL-6 stimulates metastasis by upregulating the expression on endothelial cells of adhesion molecules and by stimulating the production of growth factors [28–30]. In addition, several studies have shown that high IL-6 serum levels are associated with progression of cancer and the metastasis of cancer cells [9, 31–33]. However, in our study IL-6 was not able to differentiate advanced stages with metastasis from non-metastatic diseases.

Our study showed that PCT and IL-6 may serve as indicators of neoplastic disease, but their role as prognostic biomarkers needs to be further explored. It also showed that PCT may have a great value as a diagnostic biomarker of bacteremia or sepsis in febrile cancer patients. However the PCT level during a febrile episode should be compared in each patient to a baseline level during their non-febrile condition.

The major strength of this study was the large number of patients enrolled and the diversity of cancer types including the most common ones. One limitation our study has is enrollment of patients at different levels of their disease and therapy as 86% of them were receiving active treatment and could have responded to treatment with a decrease of the biomarkers levels. To overcome this limitation, we tried to evaluate the stage of the disease at the time of blood draws. Another study limitation is the staging system of cancer as some cancers such as hematological malignancies and brain tumors do not have a staging system and were therefore excluded from the staging analysis.

In conclusion, our results suggest a potential role for PCT and IL-6 in predicting cancer in non-febrile patients. In addition, PCT is useful in predicting progression of malignancy in non-febrile cancer patients and bacteremia or sepsis in febrile cancer patients. Further studies evaluating the role of PCT and IL-6 at baseline in cancer patients, and correlation of serum biomarkers levels with progression and regression of the tumor as well as response to chemotherapy are warranted.
